# Additive effects of warming and nitrogen addition on the performance and competitiveness of invasive *Solidago canadensis* L.

**DOI:** 10.3389/fpls.2022.1017554

**Published:** 2022-11-03

**Authors:** Guangqian Ren, Bin Yang, Miaomiao Cui, Haochen Yu, Xue Fan, Zhicong Dai, Jianfan Sun, Guanlin Li, Haiyan Zhang, Daolin Du

**Affiliations:** ^1^ Institute of Environment and Ecology, Academy of Environmental Health and Ecological Security, School of the Environment and Safety Engineering, Jiangsu University, Zhenjiang, China; ^2^ School of Agricultural Engineering, Jiangsu University, Zhenjiang, China; ^3^ School of Inspection and Testing Certification, Changzhou Vocational Institute of Engineering, Changzhou, China

**Keywords:** additive effect, nitrogen deposition, plant invasion, *Solidago canadensis*, warming

## Abstract

Changes in temperature and nitrogen (N) deposition determine the growth and competitive dominance of both invasive and native plants. However, a paucity of experimental evidence limits understanding of how these changes influence plant invasion. Therefore, we conducted a greenhouse experiment in which invasive *Solidago canadensis* L. was planted in mixed culture with native *Artemisia argyi* Levl. et Van under combined conditions of warming and N addition. Our results show that due to the strong positive effect of nitrogen addition, the temperature increases and nitrogen deposition interaction resulted in greatly enhanced species performance. Most of the relative change ratios (RCR) of phenotypic traits differences between *S. canadensis* and *A. argyi* occur in the low invasion stage, and six of eight traits had higher RCR in response to N addition and/or warming in native *A. argyi* than in invasive *S. canadensis*. Our results also demonstrate that the effects of the warming and nitrogen interaction on growth-related traits and competitiveness of *S. canadensis* and *A. argyi* were usually additive rather than synergistic or antagonistic. This conclusion suggests that the impact of warming and nitrogen deposition on *S. canadensis* can be inferred from single factor studies. Further, environmental changes did not modify the competitive relationship between invasive *S. canadensis* and native *A. argyi* but the relative yield of *S. canadensis* was significantly greater than *A. argyi*. This finding indicated that we can rule out the influence of environmental changes such as N addition and warming which makes *S. canadensis* successfully invade new habitats through competition. Correlation analysis showed that invasive *S. canadensis* may be more inclined to mobilize various characteristics to strengthen competition during the invasion process, which will facilitate *S. canadensis* becoming the superior competitor in *S. canadensis-A. argyi* interactions. These findings contribute to our understanding of the spreading of invasive plants such as *S. canadensis* under climate change and help identify potential precautionary measures that could prevent biological invasions.

## 1 Introduction

Temperature and nitrogen (N) are equally important for healthy plant growth and ecosystem balance because they provide thermal regulation and nutrition (Ren et al., 2020). Global warming and nitrogen enrichment from industrial agriculture production and pollutants have seriously altered the typical succession of plant communities induced by invasive plants (Uddin and Robinson, 2018). Evidence for the impacts of global warming or nitrogen enhancement on biological invasion at the species level is already substantial (He et al., 2011; Song, 2017; Valliere et al., 2017), but the majority of studies have focused on geographical distributions (Merow et al., 2017) or shifts in phenology and body size (Liu et al., 2017), and consider a single factor or a certain scale (Peng et al., 2019). For example, the distribution of the *Solidago canadensis* L. population can change due to significant correlations between temperature and plasticity (Li et al., 2016), and increasing global nitrogen deposition significantly favors the growth of North American *Bromus* over Chinese *Bromus* (He et al., 2011). Biological invasions are closely related to climate change (Vitousek et al., 1996; Bradley et al., 2009). But species’ varied ecological responses to environmental changes may lead to different results, and responses at a certain scale may not occur at larger scales (Hansen et al., 2001). Therefore, it is important to address how climate warming and N deposition addition interact to influence invasion success in many cases (Lu et al., 2015).

According to previous studies, global warming and increased N deposition will continue for decades to come (Galloway et al., 2004; Robinson et al., 2018), not only profoundly impacting plant growth but also influencing interspecific competition in invaded habitats (Liu et al., 2017; Song, 2017). But the complex interaction between temperature and nitrogen makes it difficult to extrapolate the impacts of this interaction on competition between native and invasive species (Jeffrey and D’Antonio, 2004; Wheeler et al., 2017). Therefore, few studies have directly examined the interacting effect of warming and N addition on competitive relationships, which would affect the invasion process (Liu et al., 2012). Competition between adjacent competitors is keen; when environmental change suppresses an adjacent superior competitor, invasion by another competitor is more likely. Conversely, when environmental change promotes an adjacent superior competitor, it will inhibit another (Liu et al., 2012). Exotic species’ superior competitive abilities are thought to be responsible for successful invasion (Pfeifer-Meister et al., 2008; Ren et al., 2022a). These competitive characteristics will impact the establishment and dispersal of exotic species (Jeffrey and D’Antonio, 2004). It is therefore important to elucidate the pattern of growth and competitive relationship between invasive and native species at different invasion levels (interspecific competition) under combined conditions of warming and N addition (van Kleunen et al., 2015; Liu et al., 2017).

Multiple environmental change drivers likely act simultaneously and influence a wide range of ecological and biogeochemical processes (Peng et al., 2019); thus, the combined effects of multiple drivers on plant invasion may be more important than the corresponding individual effects. For instance, a recent study showed that the combined effects of nitrogen addition and warming on plant performance tend to be higher than their individual effects (Cavieres et al., 2017). A key knowledge gap is whether the interaction between two drivers, defined here as interactive effects of environmental change drivers on invasive and native species, is additive or non‐additive (synergy and antagonism). Additive interactions occur when the combined effect of two or more drivers is equal to or not significantly different from the sum of the individual effects, which the impact of multiple factors on plant invasion can be inferred from single factor studies; otherwise, the interaction is either synergistic or antagonistic (Yue et al., 2019). As important elements of global environmental change, the interactive effects of flooding and nitrogen on the dominance of an exotic plant were found to be additively enhanced (Qiu et al., 2020), warming and eCO_2_ result in non-additive interactions between individual drivers for plant biomass (Mueller et al., 2016). These studies further suggest that the interactive effects of multiple environmental change drivers may vary substantially depending on the tested variables and combinations of drivers. Therefore, it is urgent to know whether the interactive effects of global warming and nitrogen deposition on specific species are additive or non-additive.

Currently, the individual, not combined, effects of environmental changes (warming and nitrogen deposition) on plant performance are commonly studied. There is a need to understand how the individual performance of invasive and native species responds differently to the more complex combinations of environmental changes that occur in nature. Further, the current comprehensive studies mainly focus on the performance of plants and did not focus on assessing the interactive effect between environmental change factors, resulting in the interactive effects of warming and nitrogen on plant invasion being additive or non-additive (synergy or antagonism) is not fully understood (Wu et al., 2020). Last, to avoid the possible bias caused by the single species selected in our experiment, and consider examining invasion on a broader scale. A systematic and rigorous approach (Integrated analysis) to quantifying the various potential behavioral of invasive species responses to the independent and interactive effects of environmental changes (warming and nitrogen deposition) is necessary (Hale et al., 2017).

Climate warming and N deposition directly affect alien invaders and native species’ growth performance through adjustment of functional trait distributions (Stige and Kvile, 2017), and indirectly affect the alien invaders by altering the invasibility of habitats (i.e. soil nutrient availability and thermal conditions) (Seebens et al., 2015). Here, we investigated how warming, N deposition, and their interaction affected the competitive relationship between invasive *S. canadensis* and native co-occurring plants and their growth. Specifically, we hypothesized that 1) N deposition addition and warming will synergistically prompt plant performance, because warming can increase plant photosynthetic activity and may accelerate rates of organic mineralization in the soil, which will increase nutrient availability (Doiron et al., 2014; Blagodatskaya et al., 2016). Wan et al. (2019) showed that N addition altered the competitive relationship between invasive *S. canadensis* and native *Pterocypsela laciniata* allowing *S. canadensis* to out-compete *P. laciniata*, and Welshofer et al. (2018) indicating climate warming (+1.8°C) favor exotic over native plant species. In that aspect, Liu et al. (2017) showed that global environmental change would generally benefit invaders more than natives. Based on this information, we put forward the second hypothesis that 2) global warming and N addition may increase the relative yield of invasive species in a microcosm. The test of these two hypotheses may have different degrees of applicability for the prediction and prevention of invasion by invasive species.

## 2 Materials and methods

### 2.1 Species selection and cultivation

**Figure 1:**
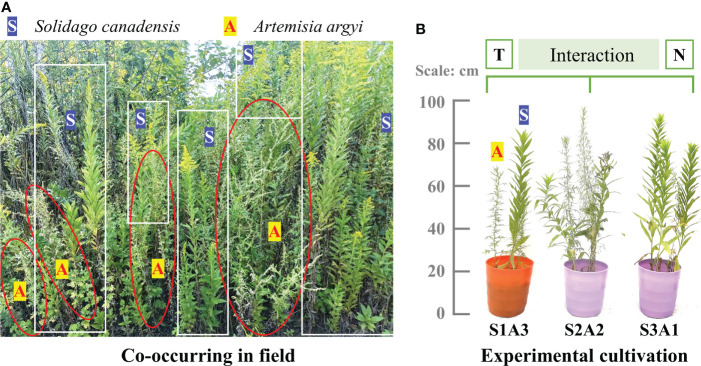
Target species: invasive *S. canadensis* (white S on blue) and native *A argyi* (red A on yellow) co-occurring in field **(A)** and cultivation in our experiment **(B)**. T, N, S, and A indicates temperature, nitrogen, invasive *S. canadensis* and native *A argyi*, respectively.

The target plant species of this research, *S. canadensis*, was introduced to China from North America in 1935. It is rapidly expanding from the Eastern coast of China into central China (Dong et al., 2017), with a faster relative growth rate, higher competitiveness, and greater adaptability than native plants (Wan et al., 2018). Co-existing species may be the first to be affected. For example, native *Artemisia argyi* Levl. et Van and invasive *S. canadensis* are both rhizomatous perennial plants with similar niches and have overlapping geographic distributions according to our pilot field investigation, especially on the Eastern coast of China (**Figure 1A**) (Ren et al., 2019). Therefore, we used these two species in our experiment.

To simulate differing degrees of invasion, seeds of invasive *S. canadensis* (hereafter referred to as S) and native *A. argyi* (hereafter referred to as A) were separately placed on the surface of the natural soil in garden pots with co-cultivation. During seed germination, adequate tap water was supplied every day. The experiment was conducted using three cultivation treatments: low degree of invasion, 1S:3A (IL, 25%); moderate degree of invasion, 2S:2A (IM, 50%); and high degree of invasion, 3S:1A (IH, 75%) (**Figure 1B**) (Si et al., 2014). The individual seeding of *S. canadensis* and *A. argyi* was also planted in pots during the experiment for calculating the competitiveness. The treatments were created by thinning to the required number of plants when the seeds germinated and had grown to about 1cm in height (Liu et al., 2018). Each treatment was replicated 20-40 times. The other details of the species selection and cultivation can be referred to our previous study Ren et al. (2022b).

### 2.2 Simulation of global warming and N deposition addition

The experiment was established on 13 May 2017, and ended on 5 September 2017. The experiment consisted of two levels of air warming treatment: warmed air temperature by 1.86°C in greenhouse and natural air temperature unwarmed adjacent to the greenhouse as the control (Rogelj et al., 2012). For nitrogen addition treatment, 12 g· m^-2^ ·yr^-1^ of nitrogen (1:1:1 ratio of KNO_3_-N: NH_4_Cl-N: Urea-N) was added as an aqueous solution, which was basically consistent with the increase rate of N deposition in the study area (Liu et al., 2013; Gu et al., 2016). Meanwhile, the control treatment received an equal volume of water in same time (Ren et al., 2019). Each of four treatments (warming (T), N input (N), the combination of warming and N input (N×T), and no warming and no nitrogen (CK)) were randomly arranged in each of four replicate blocks. The other details of the warming and N addition treatments were provided in our earlier studies Ren et al. (2020) and Ren et al. (2021).

### 2.3 Measurements

The experiment consisted of a time displacement design with 180 pots, with 4 environmental treatments (CK, T, N, N×T) × ((2 invasion degrees (LI, HI) + 2 individuals (*S. canadensis*, *A. argyi*) × 10 replicates) + (1 invasion degree (MI) × 5 replicates)) (There are two scenarios for moderate invasion degree: S2A2 and S3A3, respectively. Only S2A2 is used here, therefore, it was 5 replicates). Plants were harvested after 12 weeks of the treatments, we measured the performance traits of one plant per pot, including the height and ground diameter of the main culm. Biomass was also determined after washing and drying at 80°C for 48 h (Farrer and Suding, 2016). Chlorophyll and N content was measured with the Chlorophyll meter (YT-YD) and Elemental analyzer (CE Instruments Flash EA 1112, CE Elantech, Lakewood, NJ, USA). N absorption efficiency (NAE) was defined as total N in the plant (mg plant^−1^), and N use efficiency (NUE) was defined as the mg of plant dry weight produced per mg of N absorbed by the plant (Zhang et al., 2020). The relative yields (RY) for *S. canadensis* were calculated using the following formula (Garcia-Serrano et al., 2007): RY= Y_mixed_/(p×Y_mono_), where Y_mono_ is the yield (total biomass) of S or A in monoculture, Y_mixed_ is the yield of S or A when grown with A or S, and p is the initial proportion of species S and A in mixed culture. The higher the value of RY, the stronger the species’ competitiveness (Wu et al., 2017).

### 2.4 Statistical analyses

To minimize the species-specific difference in growth traits and better detect the effect of environmental changes, the relative change ratios (RCR) in growth traits of the indices were calculated as RCR (%) = [(Trait_T_ –Trait_ck_)/Trait_ck_]×100%, where Trait_T_ stands for the value of a given trait under experimental manipulations and Trait_ck_ represents the value under the control treatment (Ren et al., 2021). All data for the relative change in each plant growth trait were analyzed with one-sample test to test whether these relative changes differed from zero. An independent sample was also used to examine whether the relative changes in a specific trait differed between native and invasive populations.

### 2.5 Integrated analysis

We searched the ISI Web of Science (http://apps.webofknowledge.com/) using words related to “warming (temperature)”, “nitrogen (nutrition)”, and “invas*” as keywords. A total of 1940 papers published before July 2022 were assessed. Each publication was individually assessed and retained if it satisfied the following criteria: (1) it reported biomass, (2) it included simultaneous consideration of warming and nitrogen, and (3) its objective was related to plant invasion. Based on the foregoing criteria, only 9 published articles (**Appendix Table 1**) were selected. We extracted the data from the text and tables in the main body and appendices of these papers. The GetData Graph Digitizer (version 2.24, available at: http://getdata-graph-digitizer.com/) was used to extract the data from the figures. As the number of studies was limited, the amplitude of temperature and nitrogen increase was negligible, both compared with the ambient level and calculated as RCR. There were 55 invasive species data and 94 native species data were extracted and calculated. Other analysis methods were the same as described in section 2.4 Statistical analyses.

## 3 Results

We simulated different *S. canadensis* invasion degrees under combined conditions of N deposition and global warming. The results showed that Chlorophyll, biomass, NAE, NUE, and competitiveness were all sensitive to N, invasion level, and species (*p* ≤ 0.05, **Table 1**). Although diameter and height were sensitive to N and species, they were unaffected by invasion level (**Table 1**). Temperature and its interaction with N only affected the height, biomass, and NAE of the plant (*p* < 0.05, **Table 1**). NUE was affected by the interaction of temperature, N, and invasion degree (*p* < 0.01, **Table 1**). There were significant four-way interactions (temperature × N × invasion × species) on biomass, NAE, and NUE (*p* < 0.05, **Table 1**).

**Table 1:** The effects of warming (W), N addition (N), invasion level (I), species (S), and their interactions on phenotypic traits.

	Diameter	Height	Chlorophyll	Biomass	NAE	NUE	RY
Warming (W)	0.65	4.46^*^	0.13	12.71^**^	4.44^*^	0.38	0.90
Nitrogen (N)	52.59^**^	40.78^**^	14.50**	173.08^**^	130.61^**^	246.88^**^	12.26^**^
Invasion (I)	0.03	0.82	3.17*	4.96^*^	22.77^**^	20.41^**^	3.45^**^
Species (S)	45.54^**^	17.84^**^	7.66**	19.65^**^	231.45^**^	4042.77^**^	16.75^**^
T * N	0.30	4.18^*^	0.90	14.06^**^	4.10^*^	3.27	0.19
T * I	0.61	1.30	0.24	2.04	1.97	6.95^**^	0.12
T * S	1.48	1.03	0.32	7.06^*^	3.88	0.19	1.69
N * I	1.93	5.90^**^	0.86	6.23^**^	8.33^**^	6.02^**^	3.80^*^
N * S	2.51	0.24	3.77	5.68^*^	91.83^**^	124.97^**^	4.82^*^
I * S	3.98^*^	9.36^**^	2.38	110.17^**^	35.22^**^	13.81^**^	2.06
T * N * I	0.61	0.37	0.38	2.70	3.08	6.10^**^	0.49
T * N * S	0.28	0.72	0.27	3.51	3.25	2.65	2.01
T * I * S	0.77	1.62	1.29	4.80^*^	2.46	4.54^*^	0.21
N * I * S	1.47	0.45	0.67	22.36^**^	12.75^**^	5.52^**^	1.37
T * N * I * S	1.94	1.56	0.97	11.72^**^	3.96^*^	4.39^*^	1.43

** and * indicated the significant level p < 0.01 and 0.05, respectively. NAE, NUE, and RY represented nitrogen absorption efficiency, nitrogen use efficiency, and relative yield, respectively.

### 3.1 Phenotypic traits

**Figure 2:**
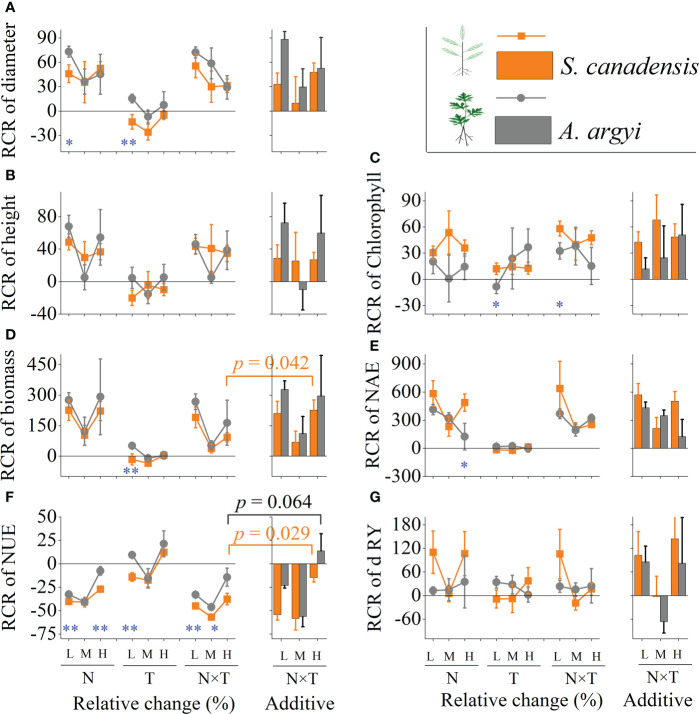
Effects of nutrient level (N) and warming (T) on the relative change ratio (RCR) of phenotypic traits of invasive *S. canadensis* and native *A. argyi* under different proportions in microcosm. Where **(A–G)** indicates the RCR of diameter, height, Chlorophyll, biomass, nitrogen absorption efficiency (NAE), nitrogen use efficiency (NUE) and relative yield (RY) of both species, respectively. Values are presented as relative change ratio (RCR) means ± SE. L, M, and H indicate low, moderated, and high invasion levels. * and ** indicate the significant level *p* < 0.1 and 0.05, respectively. The additive effect was the sum of the N addition and warming alone effects; the other difference is not presented for clarity.

The RCR results showed invasive *S. canadensis* and native *A. argyi* responded similarly to warming, nitrogen, and their interaction (**Figure 2**). At the low invasion level, warming or N addition alone resulted in the RCR of diameter being higher in *A. argyi* than in *S. canadensis* (*p* < 0.1, **Figure 2A**). The RCR of Chlorophyll was higher in *S. canadensis* than in *A. argyi* for the response to warming and interact with N addition (*p* < 0.1, **Figure 2C**). The RCR of biomass was significantly higher in *A. argyi* than *S. canadensis* only in the warming treatment (*p* < 0.05, **Figure 2D**). The RCR of NAE was higher in *S. canadensis* than *A. argyi* in response to N addition at the high invasion level (*p* < 0.1, **Figure 2E**). Five of the nine environmental treatments had greater effects on the NUE in *A. argyi* than *S. canadensis* (all *p* < 0.1, **Figure 2F**).

At the high invasion level, the value of the biomass RCR in *S. canadensis* under the warming and N addition interaction treatment was between the biomass RCR values in the warming and N addition alone treatments, while significantly lower than the sum of the individual effects, which is an antagonistic effect (*p* = 0.04, **Figure 2D**). The NUE of both species’ combined responses to warming and N addition were significantly lower than the individual responses and their sum, characteristic of the negative synergy effect (both *p* < 0.1, **Figure 2F**). All other traits including the relative yield under the interaction treatments were not significantly different from the sum of individual effects, which is the additive effect (both *p* > 0.1, **Figure 2**).

### 3.2 Correlations among phenotypic traits

Correlation analysis showed that there was a significant correlation among all the characteristics of *S. canadensis*, except between NUE and competitiveness (all |r| > 0.29, *p* < 0.05, **Table 2**). For *A. argyi*, most of the traits were significantly correlated, but Chlorophyll was only related to NAE, and competitiveness was only related to height (**Table 2**).

**Table 2:** Correlation among **phenotypic traits** of *S. canadensis* (lower triangular matrixa) and *A. argyi* (upper triangular matrixb) in mixed culture (N=35).

Traits	Diameter	Height	Chlorophyll	Biomass	NAE	NUE	RY
Diameter		0.785^**^	0.039	0.644^**^	0.686^**^	-0.614^**^	0.183
Height	0.890^**^		0.029	0.655^**^	0.723^**^	-0.483^**^	0.264^**^
Chlorophyll	0.468^**^	0.454^**^		0.192	0.346^*^	-0.141	-0.155
Biomass	0.624^**^	0.619^**^	0.386^**^		0.980^**^	-0.573^**^	-0.035
NAE	0.655^**^	0.677^*^	0.620^**^	0.961^**^		-0.697^**^	-0.071
NUE	-0.506^**^	-0.465^**^	-0.783^**^	-0.359^*^	-0.566^**^		0.040
RY	0.472^**^	0.468^**^	0.291^**^	0.458^**^	0.581^**^	-0.301	

** and * indicated the significant level p < 0.01 and 0.05, respectively. NAE, NUE, and RY represented nitrogen absorption efficiency, nitrogen use efficiency, and relative yield, respectively.

### 3.3 Warming and nitrogen deposition interaction effect

**Figure 3:**
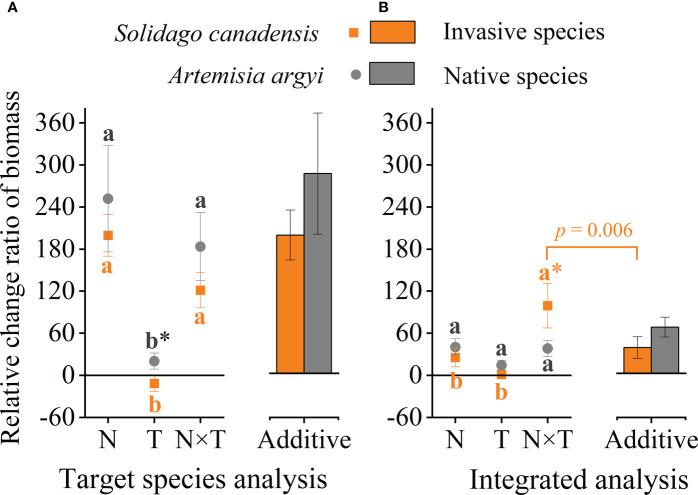
Effects of nitrogen addition (N) and warming (T) on the relative change ratio (RCR) of biomass. **(A)** target species in our experiment: invasive *S. canadensis* and native *A argyi*, **(B)** species in previous studies: invasive species and native species. Values are presented as relative change ratio means ± SE. * indicates the significance level *p* < 0.1 between invasive and native species. The additive effect was the sum of the N addition and warming alone effects. Different lowercase letters denote significant differences among treatments at the *p* = 0.05 level.

To further clarify the effects of the temperature and nitrogen interaction on invasive and native plants, we calculated the additive effect without considering the invasion degrees. The biomass RCR of *S. canadensis* was significantly lower than that of *A. argyi* under the warming treatment (*p* = 0.057), and there was no significant difference between *S. canadensis* and *A. argyi* under the N addition and its interaction with warming treatment (**Figure 3A**). Both species have a higher biomass RCR in the N addition and its interaction treatment than in the warming treatment (**Figure 3A**). The biomass RCR of both species under the interaction treatment were similar to the sum of the individual effects, indicating that warming and N addition have an additive effect on these two species (**Figure 3A**). Integrating nine papers and analysis, the biomass RCR of invasive and native plants was similar under the warming and N addition alone treatments. However, the biomass RCR for invasive plants was significantly higher than the biomass RCR for native plants under the interaction treatment (**Figure 3B**). Also, native species biomass had a similar response among the warming, N addition, and their interaction treatments, while the invasive species biomass response to the interaction treatment was significantly higher than the warming and N addition alone treatment (**Figure 3B**). The RCR of invasive species under the interaction treatment was significantly higher than the sum of the individual effects, which is a positive synergy effect. The native species under the interaction treatment were similar to the sum of the individual effects, which belong to additive effect (**Figure 3B**).

## 4 Discussion

### 4.1 The difference in phenotypic traits between *Solidago canadensis* and *Artemisia argyi*

Our results show that most of the RCR of phenotypic traits differences between *S. canadensis* and *A. argyi* occur in the low invasion stage, and six of eight traits had greater relative changes in response to N addition or warming in native *A. argyi* than in invasive *S. canadensis*. Most of these differences disappeared at medium and high levels of invasion. This may be because the allelopathic effects of invasive species on height, biomass, and community stability of native species decrease with increasing degree of invasion (Wang et al., 2016; Wang et al., 2019); the allelopathic impact of *S. canadensis* on native species may also be attenuated under increased N deposition (Wang et al., 2017; Zubek et al., 2020). Another reason may be the differences in sensitivity responses of invasive and native plants to environmental changes (Peng et al., 2019). At a high invasion level, warming and N addition have an antagonistic effect on *S. canadensis* biomass, with a negative synergy effect on the NUE of both species. This different interaction effect may be caused by the negative relationship between biomass and NUE of *S. canadensis* (**Table 2**). Therefore, the varied response of invasive and native plants to environmental changes related to the degree of invasion may provide insights into understanding the interspecific interaction and the impact of environmental variables on invasion dynamics.

*S. canadensis* and *A. argyi* biomass RCR differ significantly when responding to warming but similarly to N addition and its interaction with warming, This indicated these two species differ significantly in temperature sensitivity, but are similar in nutritional response. This may be because *S. canadensis* and *A. argyi* originated from different latitudes and have a similar change in NAE (**Figure 2**). The different leaf types between *S. canadensis* (lanceolate leaf) and *A. argyi* (pinnatipartite leaf) may be an another reason for their different response to global warming (**Figure 1**).

### 4.2 The additive effect of warming and N on invasive *Solidago canadensis*

The results obtained in this study indicate that nitrogen addition significantly increased and warming slightly decreased species’ growth, which was consistent with Jabran and Dogan (2020) who found that invasive weed species were generally negatively or neutrally affected by warming, and weeds mostly had a positive response to nitrogen application. These results may relate to soil fertility and plant phytohormones (e.g., cytokinin and auxin) improved by nitrogen addition (Tulloss and Cadenasso, 2016; Peng et al., 2019). For another, a decrease in CO_2_ assimilation (induced by the inactivation of photosynthetic enzymes) and a greater rate of maintenance respiration with the increasing temperature will ultimately reduce plant growth (Monteith and Moss, 1997; Ziska and Bunce, 1998; Jabran and Dogan, 2020).

Due to the strong positive effect of nitrogen addition, the temperature increase and nitrogen deposition interaction resulted in greatly enhanced species performance. Our results also demonstrate that the effects of the warming and nitrogen interaction on growth-related traits of *S. canadensis* and *A. argyi* were usually additive rather than synergistic or antagonistic. This conclusion suggests that the impact of warming and nitrogen deposition on *S. canadensis* can be inferred from single factor studies. Which rejects our first hypothesis that warming and N deposition addition will synergistically prompt plant performance. Source-sink relationships and resource conditions altered by environmental changes may positively influence plant performance (Peng et al., 2019). Other mechanisms may also contribute to the observed growth traits enhanced by warming and N addition, such as rapid evolution, N absorption and use efficiency, phenotypic plasticity (Merila and Hendry, 2014), and phenological development (Peng et al., 2004).

Warming and N deposition has an additive effect on both *S. canadensis* and *A. argyi*, which is different from our integrated analysis, where N addition and warming have a synergistic effect on invasive species and an additive effect on native species. This difference may be caused by the species selected and the magnitude of environmental change. With the increase from ambient to N addition nutrient levels, the biomass of invasive plants continued to increase, while the production of the native plants did not increase significantly (**Figure 3B**). As a result, although both native and invasive plants can be positively affected by the combined effects of increased temperature and nitrogen deposition, the promotion of invasive plants is greater. Similar results were also shown by Legault et al. (2018). In addition, studies have shown that warming positively affects nitrogen fixation or microorganisms involved with decomposition in soil, which makes warming energy and nitrogen deposition further interact to improve the availability of soil resources to plants, especially N (Convey et al., 2012). Many studies have shown that invasive plants have a higher positive response to nitrogen deposition than native plants (Ren et al., 2019; Zhang et al., 2022). As our experimental result showed, the RCR of NUE in invasive *S. canadensis* was significantly lower than native *A. argyi*, while this value has a significantly negative relationship with biomass. Therefore, the combined warming and N deposition show a synergistic effect on invasive plants and an additive effect on native species in integrated analysis. In addition, since none of the studies included in the integrated analysis conducted the test between cumulative effect and interaction of temperature and nitrogen, we cannot know whether there are similar interaction results in these specific studies. Therefore, more studies about multiple environmental change factors need to be explored.

### 4.3 The impact of environmental changes on competitiveness

Conti et al. (2018) have shown that the trait shifts of the alien species induced by competition can define the outcome of the invasion process at an early stage. The high relative yield of invasive plants is a key strategy for their invasion into new habitats (Leger and Rice, 2010). Accordingly, exploring shifts in the competitive relationship between invasive and co-existing species under environmental changes can be beneficial for predicting the invasion process of invasive plants or the evolutionary direction of the community (Legault et al., 2018). In our experimental study, we found that N deposition has a significant effect on relative yield, but this effect largely depends on the identity of the species and the invasion level. Warming and its interaction with N addition had no significant effects on relative yield (**Table 1**), which is contrary to the finding that global warming and nutrient deposition increased the interspecific competitiveness of the invasive plant *A. philoxeroides* (Wu et al., 2017; Zhang et al., 2017). And rejected our second hypothesis that the global environment may aggravate the relative yield of invasive species in microcosm. Meanwhile, the RCR of competitiveness in invasive *S. canadensis* and native *A. argyi* under the interaction treatments were not significantly different from the sum of individual effects, which also belong to the additive effect. These observations indicated that in our experimental results, environmental changes did not modify the competitive relationship between invasive *S. canadensis* and native *A. argyi* but the relative yield of *S. canadensis* was significantly greater than *A. argyi* (**Table 1**, **Figure 2G**). This finding indicated that the successful invasion of *S. canadensis* largely depended on its stronger competitiveness (Bradley et al., 2010), irrespective of environmental changes (Broadbent et al., 2017). In other words, we can rule out the influence of environmental changes such as N addition and warming which makes *S. canadensis* successfully invade new habitats through competition. Interspecific competition between them might be mainly caused by genetic differences such as in the target species selected in this experiment (Skovmand et al., 2018). Correlation analysis showed that the relative yield of invasive *S. canadensis* was significantly positively related to all other phenotypic traits, except NUE. The relative yield of native *A. argyi* was independent of all other indicators, except height. Indicating a traits-dependent effect that likely contributes to the competitiveness of *S. canadensis*. *S. canadensis* may also use several ecological strategies to successfully invade the native community. First, the plant’s coarse ground diameter could result in reliable and timely transportation of nutrients after they are absorbed (Kong et al., 2017). Second, a short height prevents *S. canadensis* damage from high temperatures, such as dieback (Peng et al., 2019). Third, the greater biomass production in *S. canadensis* than native *A. argyi* during the same period (not RCR) indicates the faster relative growth rate of invasive species may also play a crucial role in the invasive process (Wan et al., 2017). Therefore, we conclude that successful invasion of *S. canadensis* is largely attributed to its characteristics such as stronger competitiveness, faster relative growth rate, and resource absorption or resource use efficiency. In other words, invasive *S. canadensis* may be more inclined to mobilize various characteristics to strengthen competition during the invasion process, which will facilitate *S. canadensis* becoming the superior competitor in *S. canadensis-A. argyi* interactions.

## 5 Conclusion

Our study is the first to provide and test a case study of the positive additive effects of warming and nitrogen deposition on *S. canadensis* and *A. argyi*. Our experimental results showed that most of the differences in the relative changes of phenotypic characteristics between *S. canadensis* and *A. argyi* occur in the low invasion stage, while these differences disappeared at medium and high levels of invasion, this may be because the allelopathic effects of invasive species on native species decrease with increasing degree of invasion. Our results also demonstrate that the effects of the warming and nitrogen interaction on growth-related traits and competitiveness of *S. canadensis* and *A. argyi* were usually additive rather than non-additive. This conclusion suggests that the impact of warming and nitrogen deposition on *S. canadensis* can be inferred from single factor studies. The relative changes in relative yield between *S. canadensis* and *A. argyi* were similar when they responded to environmental changes, but the relative yield of *S. canadensis* was significantly greater than *A. argyi*. This finding indicated that interspecific competition between them might be mainly caused by genetic differences such as the target species selected in this experiment, rather than environmental changes. Correlation analysis showed that the traits-dependent effect that likely contributes to the competitiveness of *S. canadensis*. In addition, we also first conducted an integration analysis of warming and nitrogen deposition effects on invasive and native plants. The results showed that warming and N deposition combined have synergistic effects on invasive species and additive effects on native species. These results provided baseline information for our understanding of the spreading of invasive plants such as *S. canadensis* under climate change and highlighted the need to consider interspecific competition when designing strategies and policies to deal with biological invasions.

## Data availability statement

The original contributions presented in the study are included in the article/**Supplementary Material**. Further inquiries can be directed to the corresponding author.

## Author contributions

DD and ZD designed the experiment. GR, BY, XF, and MC performed the experiment, analyzed the data, and wrote the manuscript. GL, HY, HZ, and JS commented on the details of the manuscript drafts. All authors contributed to the article and approved the submitted version.

## Funding

This work was supported by the National Natural Science Foundation of China (32071521, 32271587, 31971427, 32201297, 31770446); the Carbon Peak and Carbon Neutrality Technology Innovation Foundation of Jiangsu Province (BK20220030); the Natural Science Foundation of Jiangsu Province (BK20211321); the Jiangsu Planned Projects for Postdoctoral Research Funds (2021K384C); the Priority Academic Program Development of Jiangsu Higher Education Institutions (PAPD) and the Jiangsu Collaborative Innovation Center of Technology and Material of Water Treatment.

## Conflict of interest

The authors declare that the research was conducted in the absence of any commercial or financial relationships that could be construed as a potential conflict of interest.

## Publisher’s note

All claims expressed in this article are solely those of the authors and do not necessarily represent those of their affiliated organizations, or those of the publisher, the editors and the reviewers. Any product that may be evaluated in this article, or claim that may be made by its manufacturer, is not guaranteed or endorsed by the publisher.
