# Supplementary material for: Additive effects of warming and nitrogen addition on the performance and competitiveness of invasive Solidago canadensis L
Source: Front Plant Sci. 2022 Nov 3;13:1017554. doi: 10.3389/fpls.2022.1017554 (PMC9671518; doi:10.3389/fpls.2022.1017554)
Supplement: Supplementary file 1 [file Table_1.docx]

**Appendix**

**Table 1** Warming and nitrogen effect on invasive and native species

| Warming and nitrogen deposition effect on invasive and native species | | | | | | |  |
| --- | --- | --- | --- | --- | --- | --- | --- |
|  |  | **Temperature**  **(+T: ℃)** | **Nitrogen (+N)** | **W×N** | **Invasive species** | **Native species** | |
| 1 | Ren et al. (2021) | 1.13 | 5g | I1 | *Solidago canadensis* | *Artemisia argyi* | |
|  |  | 1.86 | 12g | I2 |  |  |  |
|  |  |  |  | I3 |  |  |  |
|  |  |  |  | I4 |  |  |  |
| 2 | Colesie et al. (2020) | 13 | 14g | I1 | *Bromus erectus* | *Bromus hordeaceus* | |
|  |  | 23 |  | I2 |  |  |  |
| 3 | Luo et al. (2020) | 0.63 | 4.2 g m^−2^ | I1 | *Plantago virginica* | *NA. Plantago virginica* | |
| 4 | Lu et al. (2015) | 2 | 4g | I1 | *Alternanthera philoxeroides* | *Alternanthera sessilis* | |
| 5 | Peng et al. (2019) | 2 | 4g | I1  I2 | *CN Solidago canadensis* | *USA Solidago canadensis* | |
| 6 | Cavieres et al. (2017) | 6 | 51.8ppm | I1-alone | *Poa annua* | *Deschampsia antarctica* | |
|  |  |  |  | I1-Intrasp. |  |  |  |
|  |  |  |  | I1-Compet-*Descham* |  |  |  |
|  |  |  |  | I1-alone |  | *Colobanthus quitensis* | |
|  |  |  |  | I1-Intrasp. |  |  |  |
|  |  |  |  | I1-Compet-*Colo.* |  |  |  |
| 7 | Jabran and Dogan (2020) | 5 | 6g m2 | I1 | *Lactuca serriola L., Hordeum murinum L. and Bromus tectorum L.* |  | |
|  |  |  | 12g m2 | I2 |  |  | |
| 8 | Potter and Bowman (2020) | 4 | 2g m2 | I1 | *Bromus tectorum* |  | |
|  |  | 5 | 5g m2 | I1-invasion | *Alliaria petiolata* | *Acer rubrum* | |
|  |  |  |  | I1-no invasion |  |  | |
| 9 | Zhang et al. (2015) | 1.71 | 10 g m^-2^ | I1-Ab-2006 |  | *Graminoid species+Nongraminous forbs* | |
|  |  | 0.58 |  | I1-Ab-2007 |  |  |  |
|  |  |  |  | I1-Ab-2008 |  |  |  |
|  |  |  |  | I1-Ab-2009 |  |  |  |
|  |  |  |  | I1-Bb-2006 |  |  |  |
|  |  |  |  | I1-Bb-2007 |  |  |  |
|  |  |  |  | I1-Bb-2008 |  |  |  |
|  |  |  |  | I1-Bb-2009 |  |  |  |
|  |  |  |  |  |  |  | |
| 10 | **This study (Our)** | **1.86** | **12g** | **I1** | ***Solidago canadensis*** | ***Artemisia argyi*** | |

**Reference**

Cavieres, L.A., Sanhueza, A.K., Torres-Mellado, G., and Casanova-Katny, A. (2017). Competition between native Antarctic vascular plants and invasive *Poa annua* changes with temperature and soil nitrogen availability. *Biological Invasions* 20(6)**,** 1597-1610. doi: 10.1007/s10530-017-1650-7.

Colesie, C., Stangl, Z.R., and Hurry, V. (2020). Differences in growth-economics of fast vs. slow growing grass species in response to temperature and nitrogen limitation individually, and in combination. *BMC Ecol* 20(1)**,** 63. doi: 10.1186/s12898-020-00333-3.

Jabran, K., and Dogan, M.N. (2020). Elevated CO_2_, temperature and nitrogen levels impact growth and development of invasive weeds in the Mediterranean region. *J Sci Food Agric* 100(13)**,** 4893-4900. doi: 10.1002/jsfa.10550.

Lu, X.M., Siemann, E., Wei, H., Shao, X., and Ding, J.Q. (2015). Effects of warming and nitrogen on above- and below-ground herbivory of an exotic invasive plant and its native congener. *Biological Invasions* 17(10)**,** 2881-2892. doi: 10.1007/s10530-015-0918-z.

Luo, X., Zheng, Y., Xu, X., Xiao, R., Guo, H., and Hui, D. (2020). The impacts of warming and nitrogen addition on competitive ability of native and invasive populations of Plantago virginica. *Journal of Plant Ecology* 13(6)**,** 676-682. doi: 10.1093/jpe/rtaa055.

Peng, Y., Yang, J.X., Zhou, X.H., Peng, P.H., Li, J.J., Zhang, S.M., et al. (2019). An invasive population of *Solidago canadensis* is less sensitive to warming and nitrogen-addition than its native population in an invaded range. *Biological Invasions* 21(1)**,** 151-162. doi: 10.1007/s10530-018-1812-2.

Potter, T.S., and Bowman, W.D. (2020). Testing invasion filters for the alpine: the roles of temperature, nitrogen deposition and soil. *Biological Invasions* 22(6)**,** 1889-1901. doi: 10.1007/s10530-020-02225-5.

Ren, G.Q., Zou, C.B., Wan, L.Y., Johnson, J.H., Li, J., Zhu, L., et al. (2021). Interactive effect of climate warming and nitrogen deposition may shift the dynamics of native and invasive species. *Journal of Plant Ecology* 14(1)**,** 84-95. doi: 10.1093/jpe/rtaa071.

Zhang, T., Guo, R., Gao, S., Guo, J., and Sun, W. (2015). Responses of plant community composition and biomass production to warming and nitrogen deposition in a temperate meadow ecosystem. *PLoS One* 10(4)**,** e0123160. doi: 10.1371/journal.pone.0123160.
